# Longitudinal association of premature atrial contractions with atrial fibrillation and brain ischemia in people with type 2 diabetes: The Hoorn Diabetes Care System cohort

**DOI:** 10.1016/j.ahjo.2023.100321

**Published:** 2023-09-06

**Authors:** Peter P. Harms, Jelle C.L. Himmelreich, Marieke T. Blom, Joline W.J. Beulens, Giel Nijpels, Petra Elders, Wim A.M. Lucassen

**Affiliations:** a Amsterdam UMC location Vrije Universiteit Amsterdam, General Practice Medicine, Boelelaan 1117, Amsterdam, the Netherlands; b Amsterdam UMC location University of Amsterdam, General Practice Medicine, Meibergdreef 9, Amsterdam, the Netherlands; c Amsterdam UMC location Vrije Universiteit Amsterdam, Epidemiology and Data Science, Boelelaan 1117, Amsterdam, the Netherlands; d Amsterdam Public Health, Personalized Medicine, and Health Behaviors & Chronic Diseases, Amsterdam, the Netherlands; e Amsterdam Cardiovascular Sciences, Heart Failure & Arrhythmias, and Diabetes & Metabolism, Amsterdam, the Netherlands; f Julius Center for Health Sciences and Primary Care, University Medical Center Utrecht, Utrecht, the Netherlands

**Keywords:** Premature atrial contractions, Atrial fibrillation, Transient ischemic attack, Stroke, Type 2 diabetes

## Abstract

**Background:**

Premature atrial contractions (PACs) are potential markers for imminent onset of both atrial fibrillation (AF) and brain ischemia (BI; transient ischemic attack [TIA] or ischemic stroke). We investigated the association of PACs with incident AF and BI events separately, and of incident AF with BI events in people with type 2 diabetes (T2D) without pre-existing AF or cerebrovascular disease.

**Methods:**

A prospective longitudinal study of 12,242 people with T2D without known AF or cerebrovascular disease from the Hoorn Diabetes Care System cohort. Annual measurements (1998–2018) included cardiovascular risk factors, over 85,000 ECGs, and self-reported cardiovascular events. We assessed the association of PACs with incident AF and BI events and of incident AF with BI events using time-dependent Cox-regression models, adjusted for time-varying cardiovascular risk factors and medication use (Hazard Ratios with 95%CIs).

**Results:**

The baseline mean age was 62.2 ± 11.9 years. During a median follow-up of 7.0 (IQR 3.4–11.0) years, 1031 (8.4 %) participants had PACs, and 566 (4.6 %) had incident AF at any of the median 6 (IQR 3–10) annual ECG recordings. BI events occurred in 517 (4.2 %) people (304 TIAs, 213 ischemic strokes). After adjustment, PACs were associated with incident AF (Hazard Ratio, 1.96 (95%CI, 1.53–2.50)), but not with overall BI events (1.09 (0.76–1.56)), or with TIA (0.91 (0.57–1.46)) or ischemic stroke (1.50 (0.88–2.54)) separately. AF was not associated with BI events (0.95 (0.55–1.63)).

**Conclusions:**

In people with T2D without a history of AF or BI events, PACs are associated with a two-fold increased risk of incident AF.

## 1 Introduction

People with type 2 diabetes (T2D) have an approximately 35 % higher risk of developing atrial fibrillation (AF) than people without T2D [1]. Whereas T2D confers an up to two-fold higher risk of stroke because the involved metabolic alterations catalyze vascular arteriosclerosis and thrombogenesis [2], AF further increases the risk of brain ischemia (BI; transient ischemic attack [TIA] or ischemic stroke) [3,4]. When AF is diagnosed, prescription of anti-thrombotic medication reduces the excess risk of stroke by a fifth to two-thirds [5]. Therefore, early detection of AF in people with T2D is likely to decrease the burden of BI events in this high-risk group [3].

Periodic electrocardiographic (ECG) screening for cardiovascular risk assessment in people with T2D is currently recommended only for those with concomitant hypertension or suspected cardiovascular disease, and screening for AF is only recommended in people aged 65 years and older [6].

However, ECG markers might enable early recognition of people at increased risk of developing AF and facilitate targeted screening [7]. Potential ECG markers for AF are premature atrial contractions (PACs). Until recently, these ectopic beats were regarded as benign and clinically insignificant findings [8]. Recent prospective studies in the general population report that PACs are associated with an up to five-fold higher risk of AF, and a one-and-a-half-fold higher risk of ischemic stroke [[9], [10], [11], [12], [13], [14], [15]]. Additionally, a meta-analysis demonstrated that frequent PACs are associated with incident AF, BI or all-cause mortality [16], and the association was more pronounced in sub-populations at higher risk of cardiovascular disease. The association of PACs with BI could be both independent from and (partially) mediated by AF.

In people with T2D, PACs and AF are common findings [17], and the association of several other ECG abnormalities with cardiovascular events did not differ across subgroups by age, hypertension or estimated cardiovascular risk [18]. Moreover, studies that analyzed repeated ECG recordings during follow-up reported stronger associations with the outcomes, than studies that only analyzed baseline ECGs [[19], [20], [21]], indicating that repeated ECG recordings could provide additional insight into the value of PACs in assessing risk of AF. However, no studies investigated the association of PACs with AF or BI events in people with T2D, or have considered incident PACs after the baseline measurement.

Therefore, we aimed to investigate the association of PACs with incident AF and BI events separately and of incident AF with BI events in people with T2D without pre-existing AF or cerebrovascular disease.

## 2 Materials and methods

### 2.1 Design and population

The Hoorn Diabetes Care System (DCS) cohort consists of people with T2D from the West-Friesland region in The Netherlands. Details of the cohort have been described previously [22]. Initiated in 1998 as a prospective dynamic cohort of people with T2D, General Practitioners (GPs) could refer their T2D patients to the DCS center for annual follow-up measurements and treatment. From 2010, all people with newly diagnosed T2D in the West-Friesland region were referred to the DCS center. In 2018, the DCS cohort consisted of 14,604 people with T2D, approximately 95 % of all people with T2D from the catchment region. At the DCS center, trained research personnel annually examined participants according to standard operating procedures, including anthropometrics, blood pressure, blood samples, an ECG recording, and documentation of medication use and self-reported cardiovascular events.

### 2.2 Study sample

**Fig. 1:**
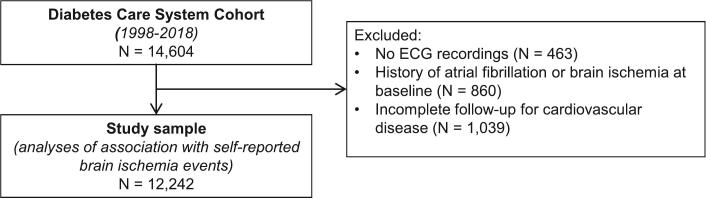
Study participant inclusion/exclusion flowchart.

We used the annual examination data from the period 1998–2018. Of the 14,604 people in the DCS cohort with at least one annual examination, we excluded 463 because they did not have any ECG recorded. We also excluded 860 people with a history of AF or ischemic cerebrovascular disease at baseline, defined as AF on ECG, or self-reported transient ischemic attack (TIA) or ischemic stroke at the participant's first ECG recording after entry into the DCS cohort. We excluded a further 1039 people because they had incomplete follow-up for cardiovascular events, including cerebrovascular events. The remaining 12,242 (83.8 %) participants were included in the analyses (Fig. 1).

### 2.3 PACs and AF

During the annual examinations trained personnel recorded a standard 10-s 12‑lead resting ECG. One trained examiner subsequently evaluated and coded all ECGs according to the Minnesota Classification system [23]. In a random sample (n = 60), the coding was compared with the coding of two independent cardiologist, showing a specific agreement for atrial arrhythmic abnormalities of between 0.87 (95%CI, 0.77 to 0.93) and 0.97 (95%CI, 0.95 to 0.99) [24]. In 2020, a consistency over time analysis in which the examiner blindly recoded random ECGs from both 2002 (n = 60) and 2016 (n = 60), showed a specific agreement for atrial arrhythmic abnormalities between 0.97 (95%CI, 0.92 to 0.99) and 0.99 (95%CI, 0.97 to 1.00) (unpublished results).

We defined PACs as the presence of one or more atrial or junctional premature beats in any of the recorded complexes (MC codes 8-1-1 and 8-4-2), and AF as persistent or intermittent atrial fibrillation or atrial flutter (MC codes 8-3). The exact description according to the Minnesota Classification is given in the supplementary material, Table S1.

### 2.4 BI events

At the annual DCS examinations, cardiovascular morbidity was assessed through self-report and was classified as TIA, stroke, myocardial infarction, angina pectoris, peripheral artery disease, heart failure, cardiac arrest and arrhythmia. The self-reported cardiovascular events were validated against the electronic patient registration of the regional hospital in a random sample of 453 participants. The sensitivity and specificity were 86 % and 90 %, and positive and negative predictive values were 90 % and 87 %, respectively [22].

We defined BI as TIA or ischemic stroke and used the self-reported TIA and stroke events to record BI events. Stroke was considered ischemic if subsequently anti-coagulants were prescribed, defined as (new) use of anti-thrombotic medication (ATC codes B01) reported at the first follow-up examination within one and a half year after the stroke date.

Deceased participants were registered every six months via the national population registry. The cause of death was determined from GP and regional hospital records and coded according to the International Classification of Diseases, Injuries and Causes of Death, ninth revision (ICD-9). For death due to TIA and ischemic stroke, we used ICD-9 codes 435 and 434, respectively.

### 2.5 Covariables

We recorded sex, date of birth, date of T2D diagnosis and educational level at entry into the DCS cohort through self-report. The highest attained educational level was classified as either low (elementary school or less), middle (lower vocational), or high (general secondary education, higher vocational or university). All other variables were assessed annually.

Smoking behavior was classified as: never, former smoker, and current smoker. Body mass index (BMI) was calculated by dividing body weight by height squared (both measured barefooted and unclothed). Systolic and diastolic blood pressure was measured twice (3 min apart) at the upper right arm after 5 min of rest with participants in a seated position, using an automatic oscillometric digital blood pressure device (Welch Allyn ProBP 3400, Skaneateles Falls, New York, USA). We determined fasting glucose level (in fluorinated plasma with the UV test using hexokinase), glycated hemoglobin (HbA1c, turbidimetric inhibition immunoassay for whole haemolysed EDTA-blood), total cholesterol, high-density lipoprotein (HDL) cholesterol, triglyceride, creatinine level, and urinary albumin (measured immunoturbidimetricly) and creatinine from overnight fasting blood or urine samples using standard enzymatic methods (Cobas c501 analyzer, Roche Diagnostics, Mannheim, Germany). Cholesterol ratio was determined by dividing the total cholesterol by HDL-cholesterol. We calculated low-density lipoprotein (LDL) cholesterol using the Friedewald formula [25]. The estimated glomerular filtration rate (eGFR) was calculated using the Chronic Kidney Disease Epidemiology Collaboration (CKD-EPI) equation [26], and the urinary albumin creatinine ratio (UACR) by dividing albumin in mg/l by creatinine in mmol/l.

We obtained information on medication use by inspecting dispensing labels, registering the name of the drug, prescribed quantity, dosage, and the Anatomical Therapeutic Chemical classification code. The use of glucose-lowering medication (A10A or A10B codes) was classified as: no medication, oral medication only, insulin use only, and combined oral and insulin use. Anti-thrombotic (B01 codes), anti-hypertensive (C02, C03A, C03B, C03E, C03DA, C07, C08, or C09 codes) and lipid-modifying (C10 codes) medication use were classified as: no or yes.

Hypertension was defined as elevated blood pressure (systolic>140 mmHg or diastolic>90 mmHg) and/or anti-hypertensive medication use, dyslipidemia as elevated LDL-cholesterol (>2.4 mmol/l) and/or lipid lowering medication use. We categorized eGFR as: normal or high (>90 ml/min), mildly decreased (60–90 ml/min), moderately decreased (30–60 ml/min) or severely decreased (<30 ml/min). We categorized albuminuria as: normal to mild (<3 mg/mmol), moderate (3–30 mg/mmol) or severe (>30 mg/mmol).

### 2.6 Statistical analyses

Baseline was defined as the first study visit with available 12‑lead ECG. We calculated baseline characteristics for the total study sample and for participants with and without prevalent PACs at baseline and with and without PACs during any study visit, reporting means with standard deviations (SD), medians with interquartile range (IQR) or percentages as appropriate.

We calculated the prevalence of PACs at baseline and during the entire follow-up, respectively, and the incidence of AF stratified by prevalent PACs at baseline or at any examination. We reported the median follow-up time, the median number of ECG recordings, and the number of observed TIA and/or ischemic stroke events. In addition, we calculated incidence rates and plotted incidence curves for AF and BI events stratified by PACs or AF at any study visit.

During follow-up, 3193 (3.6 %) ECG measurements were missing. In case of missing ECG data, we imputed PAC and AF status with the last value carried forward method until the next non-missing value. The missing measurements for the covariables were calculated stratified by consecutive annual examination (Supplementary material, Fig. S2). The total proportion of missing values for all covariables over the 20 year follow-up period was only 1.7 % (34,385/2,041,710), therefore we excluded the missing values pair-wise.

We used time-dependent Cox-regression models for repeated measurements to evaluate the association of PACs with incident AF and BI events (TIA and ischemic stroke) and of incident AF with BI events, computing hazard ratios (HRs) with 95 % confidence intervals (95%CI). To adjust for confounding models were built using a step-wise approach: model 1, unadjusted; model 2, adjusted for age and sex; model 3, additionally adjusted for smoking behavior, BMI, systolic blood pressure, HbA1c, and TC/HDL–C ratio; model 4, additionally adjusted for education, eGFR, UACR, glucose-lowering medication use, lipid-modifying medication use, and antihypertensive medication use.

PACs and AF were modelled as irreversible meaning that a participant's status remained positive once a PAC or AF was detected on an annual ECG, even when PAC or AF was not detected at subsequent annual visit ECGs. Covariables were modelled as time-varying (age, smoking behavior, BMI, systolic blood pressure, HbA1c, TC/HDL–C ratio, eGFR, UACR, antihypertensive medication, glucose-lowering medication and lipid-modifying medication), or as time-constant (sex, and education).

Follow-up duration was defined as the time from baseline to first AF or BI event (depending on the outcome of the analysis), last contact date (in case of loss to follow-up) or date of death (if participants died of a cause other than BI)

whichever occurred first.

In all analyses, significance was assessed at the p < 0.05 (two-sided) or 95%CI level. Analyses were performed using SPSS version 26 (IBM corporation, New York, USA) [27], and R (studio) version 4.0.3 (R foundation for statistical computing, Vienna, Austria) [28] with the R packages *haven* (2.3.1) [29], *epiR* (2.0.19) [30], *survival* (3.2-7) [31], *survminer* (0.4.8) [32], and *ggplot2* (3.3.2) [33].

## 3 Results

### 3.1 Baseline characteristics

At baseline, the mean age of the study population was 62.2 ± 11.9 years, median T2D duration was 0.6 (IQR, 0.2–3.2) years, and 53.1 % was male (Table 1). Compared to participants without PACs, participants with PACs were older, more frequently male, former smoker and lower educated, had higher blood pressure, lower eGFR, higher UACR, and used more insulin, antithrombotic, antihypertensive and lipid-lowering medication. These differences were more prominent in people with PACs at baseline compared to people with PACs at any examination during follow-up. Additionally, participants with PACs at baseline and/or during follow-up, had higher incidence of AF during follow-up.

**Table 1:** Characteristics of the study population at baseline, stratified by prevalence of PACs at baseline, or at any examination.

Characteristic	Total	Prevalence at baseline		At any examination	
		No PACs	PACs	No PACs	PACs
Number	12,242^a^	12,074	168	11,211	1031
Incident AF during follow-up	4.6 %	4.6 %	9.5 %	4.0 %	11.3 %
Age (years)	62.2 ± 11.9	62.1 ± 11.9	70.6 ± 10.3	61.9 ± 12.0	66.1 ± 9.8
Men (%)	53.1 %	53.0 %	60.7 %	52.9 %	56.1 %
T2D duration (years)	0.6 (0.2–3.2)	0.6 (0.2–3.2)	0.9 (0.2–3.3)	0.6 (0.2–3.2)	0.6 (0.2–3.1)
Educational level (%)					
Low	43.6 %	43.5 %	50.0 %	42.9 %	50.7 %
Middle	40.7 %	40.7 %	43.0 %	41.3 %	34.7 %
High	15.7 %	15.8 %	7.0 %	15.8 %	14.6 %
Smoking behavior (%)					
Never	39.3 %	39.3 %	34.4 %	39.3 %	38.6 %
Former	39.1 %	39.0 %	47.5 %	38.6 %	44.0 %
Current	21.7 %	21.7 %	18.1 %	22.0 %	17.4 %
BMI (kg/m^2^)	30.3 ± 5.5	30.3 ± 5.5	29.6 ± 5.0	30.3 ± 5.5	29.8 ± 5.0
SBP (mmHg)	142.9 ± 20.9	142.8 ± 20.9	149.7 ± 23.4	142.5 ± 20.9	147.0 ± 20.6
DBP (mmHg)	80.9 ± 10.0	80.9 ± 10.0	77.7 ± 10.5	80.9 ± 10.0	81.0 ± 10.6
HbA1c (%)	6.7 (6.2–7.7)	6.7 (6.2–7.7)	6.6 (6.1–7.5)	6.7 (6.2–7.7)	6.7 (6.2–7.6)
HbA1c (mmol/mol)	50.0 (44.0–60.7)	50.0 (44.0–60.7)	48.6 (43.2–58.2)	50.0 (44.0–61.0)	49.7 (44.0–59.6)
Fasting glucose (mmol/l)	7.9 (7.0–9.2)	7.9 (7.0–9.2)	7.5 (6.8–8.4)	7.9 (7.0–9.2)	7.8 (6.8–9.0)
Total cholesterol (mmol/l)	5.1 ± 1.2	5.1 ± 1.2	4.9 ± 1.1	5.1 ± 1.2	5.1 ± 1.1
LDL cholesterol (mmol/l)	3.0 ± 1.0	3.0 ± 1.0	2.8 ± 0.9	3.0 ± 1.0	3.1 ± 1.0
HDL cholesterol(mmol/l)	1.2 ± 0.3	1.2 ± 0.3	1.3 ± 0.4	1.2 ± 0.3	1.3 ± 0.4
TC/HDL ratio	4.2 (3.4–5.2)	4.2 (3.4–5.2)	3.9 (3.2–4.7)	4.2 (3.4–5.2)	4.1 (3.3–5.1)
Triglycerides (mmol/l)	1.6 (1.2–2.3)	1.6 (1.2–2.3)	1.4 (1.1–1.9)	1.6 (1.2–2.3)	1.6 (1.1–2.1)
eGFR (ml/min)	80.2 ± 18.6	80.4 ± 18.6	69.9 ± 18.9	80.6 ± 18.7	76.1 ± 16.9
UACR (mg/mmol)	0.6 (0.4–1.4)	0.6 (0.4–1.4)	0.7 (0.4–2.1)	0.6 (0.4–1.4)	0.7 (0.4–1.5)
Glucose-lowering medication use (%)					
No medication	30.7 %	30.7 %	31.0 %	30.8 %	30.4 %
Oral only	59.8 %	59.9 %	54.2 %	59.7 %	60.9 %
Insulin only	4.8 %	4.7 %	8.9 %	4.8 %	4.2 %
Oral & insulin	4.7 %	4.7 %	6.0 %	4.7 %	4.6 %
Anti-thrombotic medication (%)	22.5 %	22.3 %	36.9 %	22.2 %	25.6 %
Anti-hypertensive medication (%)	55.6 %	55.4 %	69.6 %	55.3 %	58.7 %
Lipid-lowering medication (%)	39.7 %	39.5 %	49.4 %	39.8 %	38.5 %
Hypertension (%)	74.2 %	74.1 %	85.1 %	73.6 %	80.9 %
Dyslipidemia (%)	91.9 %	91.8 %	92.6 %	91.8 %	92.8 %
eGFR categories (%)					
normal or high (>90)	31.7 %	32.0 %	14.1 %	32.8 %	19.7 %
Mildly decreased (60–90)	54.8 %	54.7 %	58.9 %	53.8 %	65.2 %
Moderately decreased (30–60)	13.0 %	12.8 %	25.8 %	12.8 %	14.5 %
Severely decreased (<30)	0.5 %	0.5 %	1.2 %	0.5 %	0.6 %
Albuminuria					
Normal to mild (UACR <3)	86.2 %	86.3 %	80.5 %	86.2 %	86.4 %
Moderate (UACR 3–30)	11.8 %	11.7 %	15.1 %	11.8 %	11.3 %
Severe (UACR >30)	2.1 %	2.0 %	4.4 %	2.0 %	2.3 %

Data are presented as mean ± SD, median (IQR), or proportion %(n/N). Baseline was defined as the first annual examination with an ECG recording after entry into the DCS cohort.

PACs, premature atrial contractions; AF, atrial fibrillation; BMI, body mass index; SBP, systolic blood pressure; DBP, diastolic blood pressure; HbA1c, hemoglobin A1c; LDL, low density lipoprotein; HDL, high density lipoprotein; TC, total cholesterol; eGFR, estimated glomerular filtration rate; UACR, urinary albumin creatinine ratio; CVD: cardiovascular disease.

a Approximately 90 % of the study population had European ancestry.

### 3.2 Follow-up for PACs, AF, and BI events

During a median follow-up of 7.0 (IQR 3.4–11.0) years, 1031 (8.4 %) of the participants had PACs at any study ECG, and 566 (4.6 %) had incident AF at any of the median 6 (IQR 3–10) annual ECG recordings. BI events occurred in 517 (4.2 %) people, of which 304 were TIA, and 213 ischemic strokes.

**Fig. 2:**
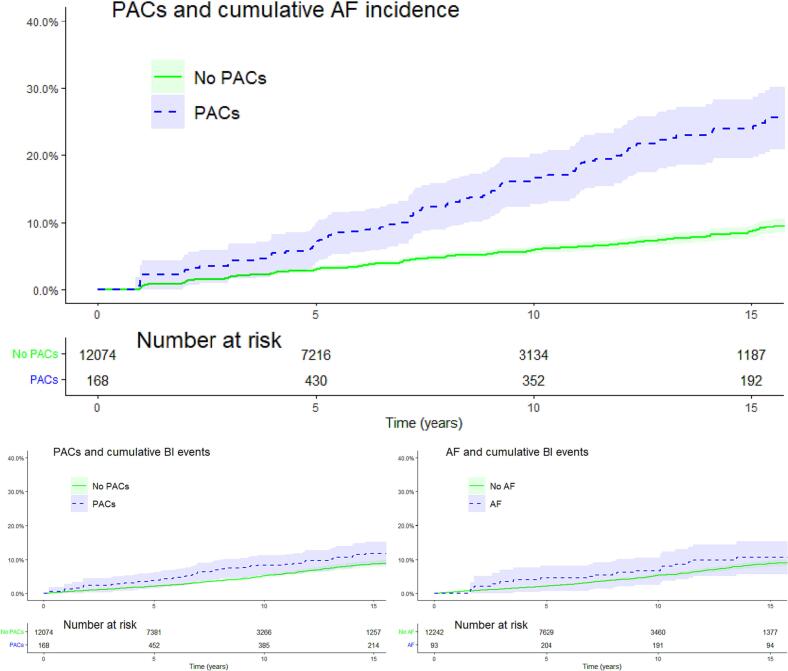
Cumulative incidence of AF and BI, stratified by presence of PACs or AF on study ECG with confidence intervals (shaded areas). PAC: Premature atrial contractions, AF: Atrial Fibrillation, BI: Brain Ischemia. Note: The curves are based on instantaneous hazard functions that change over time, and therefore in time-dependent analyses depict the cumulative events for (hypothetical) participants with an ECG abnormality status that is time-constant over the whole follow-up period.

The crude incidence rate of AF per 1000 person-years (95%CI) was more than three-fold higher in participants with PACs (20.4 (16.8–24.5)) on any previous study ECG, compared to participants without PACs (6.1 (5.6–6.6)) (supplementary material, Table S3). The crude incidence rate of BI events (TIA or ischemic stroke) per 1000 person-years was a marginally significant one-and-a-half-fold higher in participants with PACs (8.0 (5.9–10.7)), compared to participants without PACs (5.3 (4.8–5.8)), and a statistically insignificant one-and-a-half-fold higher in participants with AF (7.9 (4.9–12.0)), compared to participants without AF (5.4 (4.9–5.9)). The cumulative incidence curves provided similar results for the difference between participants with and without PACs, albeit somewhat attenuated. There was no significant difference in cumulative incidence of BI events between participants with and without AF (Fig. 2).

### 3.3 Association between PACs, AF and BI

**Fig. 3:**
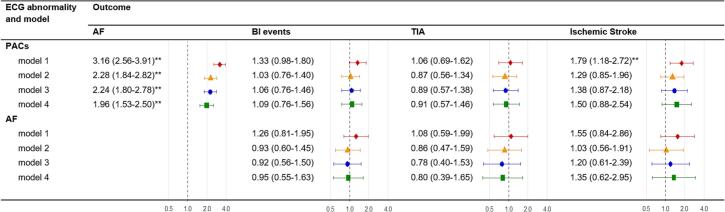
Hazard ratios with 95 % confidence intervals for AF and BI events from time-dependent Cox-regression models. PACs: Premature atrial contractions, AF: Atrial Fibrillation, BI: Brain Ischemia, TIA: Transient ischemic attack. Model 1, unadjusted. Model 2, adjusted for age and sex. Model 3, adjusted for age, sex, smoking behavior, BMI, systolic blood pressure, HbA1c, and TC/HDL–C ratio. Model 4, adjusted for age, sex, smoking behavior, BMI, systolic blood pressure, HbA1c, and TC/HDL–C ratio, education, eGFR, UACR, glucose-lowering medication, lipid-modifying medication, and antihypertensive medication use. * Significant at the p < 0.05 level (two-sided). ** Significant at the p < 0.01 level (two-sided).

After adjustment for all covariables in model 4, PACs on any previous study ECG were associated with incident AF (HR, 1.96 (95%CI, 1.53–2.50)), but not with BI events (1.09 (0.76–1.56)), or with TIA (0.91 (0.57–1.46)) or ischemic stroke (1.50 (0.88–2.54)) separately (Fig. 3). However, the HRs for TIA of just below one were consistently of the opposite direction and lower in magnitude compared to the HRs for ischemic stroke of approximately one-and-a-half. Lastly, AF on ECG after baseline was not associated with BI events (0.95 (0.55–1.63)), TIA (0.80 (0.39–1.65)) or ischemic stroke (1.35 (0.62–2.95)).

## 4 Discussion

### 4.1 Principal findings

This study showed an approximately two-fold increased incidence of AF in people with T2D with PACs, compared to people with T2D without PACs. However, we observed no increased risk for overall BI events, or for TIA or ischemic stroke separately in people with T2D with PACs or AF.

### 4.2 Comparison to previous work

Kamel and colleagues (2016) proposed that both PACs and AF should be seen as signs of atrial cardiomyopathy, and that PACs may be independently related to clinical outcomes, while AF is an already clinically established (thrombogenic) variant [34]. In line with this novel framework, previous studies conducted in general populations reported associations of PACs with AF: a three-fold to five-fold increased risk of AF (over a 14-year follow-up period) in the Japanese IPHS cohort [9], a nearly three-fold increased risk of AF in the European Copenhagen Holter Study [10], an almost two-fold increased risk of AF in the North-American REGARDS cohort [11], and a roughly one-and-a-half-fold increased risk of AF in the North-American CHS and ARIC cohorts [12]. These risks are similar to the two-fold increased incidence of AF in people with T2D and PACs in our current study. This finding suggests that the association between PACs and AF is not modified by T2D, and somewhat contradicts the more pronounced association in other high-risk populations observed by a recent meta-analysis [16]. The higher risk found in the Japanese IPHS cohort maybe explained by the different ancestry, the on average twice as long follow-up or the six times lower overall incidence rate of AF in that study, possibly a result of not counting atrial flutter as AF. Our study is the first to confirm the association of PACs with AF in people with T2D.

Our study does not indisputably confirm previously reported associations of PACs with a roughly one-and-a-half-fold increased risk of ischemic stroke or stroke mortality in the IPHS, the Copenhagen Holter study, the ARIC, and the REGARDS cohorts [9,[13], [14], [15]]. Despite the similar magnitude of the hazard ratio point estimates across our analysis models, the association between PACs and ischemic stroke was only significant in the unadjusted model, not in the adjusted models. In our analysis, there was also no association of PACs with TIAs. Moreover, in this study we report a similar absence of association for AF with ischemic stroke and/or with TIA, despite AF being a known cause of BI events. A plausible explanation for these remarkable results is that in the Hoorn DCS cohort incident AF on one of the annual ECG recordings commonly led to the initiation of adequate thrombosis prophylactic medication and (intensified) cardiovascular risk management according to the Dutch College of General Practitioners guidelines [35]. This sort of timely treatment might well have effectively reduced the excess risk of BI events [5].

PACs have been called signs of atrial cardiomyopathy that convey a risk of stroke both independent from and through its association with AF [34]. Secondary analyses from previous studies hinted that AF could indeed be a mediator of the association between PACs and cardiovascular risk factors, stroke and mortality [12,36]. Our analyses support this as the lack of a significant association with BI events for both AF (likely due to treatment following incident AF on annual ECG) and PACs in combination with the strong association of PACs with AF suggest that any risk for BI associated with PACs does run through its association with AF. Conversely, if the association of PACs with ischemic stroke was insignificant because the power of the secondary analyses was slightly too low, PACs might be associated with ischemic stroke both through AF and subsequent thromboembolism and independently from AF via arteriosclerotic small vessel disease. Therefore, a (sufficiently powered) proper mediation analysis of the association between PACs, AF and BI events would be of interest. However, we were unable to perform this mediation analysis because methods for mediation analysis with time-dependent survival analysis have not yet been established.

### 4.3 Clinical relevance

There is a need for low-cost markers that help physicians decide which people with T2D would benefit from more stringent follow-up or targeted screening for risk of cardiovascular events. This need will not likely decrease in the foreseeable future because the prevalence of T2D is increasing worldwide [37]. Our findings indicate that a PAC on 12‑lead ECG could constitute such a marker for AF, and that encountering PACs on an ECG in people with T2D should not be regarded as merely a benign finding but should lead to awareness of a higher risk of incident AF. Moreover, our findings support current guidelines that recommend periodic ECG recording in people with T2D [6] and further research into the added value of ECG-aided clinical screening models for people with T2D. Additionally, they underwrite previous calls for more stringent follow-up in those with PACs on ECG [16], specifically in people with T2D who are at increased risk of AF and ischemic stroke [4].

Still, from our observational study it remains unclear if PACs are useful markers in AF screening to ultimately lower incidence of BI events. We observed no increased risk for BI events associated with the detection of PACs. However, we also observed no increased risk for BI events associated with AF, while AF is known to increase the risk of ischemic stroke in older people with T2D [4]. This indicates that routine care after AF diagnosis on annual ECG recordings mitigated the excess risk of ischemic stroke associated with AF in the Hoorn DCS cohort of people with T2D. This is an argument in favor of early AF detection, with PACs as a potential marker to facilitate timely AF detection. Nonetheless, prospective trials that investigate PACs as markers for more stringent screening for AF with extensive follow-up for BI events would be required for definitive recommendations.

### 4.4 Strengths and limitations

This study has a number of strengths. First, the DCS is a large unselected population-based cohort with real-world data of people with T2D in primary care. Second, the DCS dataset contains measurements of over 85,000 annual study visits from over 15 years of follow-up with detailed information and a high level of completeness. Third, time-varying analyses closely resemble clinical practice in which individuals' risk profiles change over time, compared to classical time-to-event analyses that assess exposures and confounders only at baseline. Finally, PACs and AF were assessed with annual study ECGs, enabling an assessment of the potential for incident AF detection with periodic screening ECG in people with T2D.

A limitation of this study is the use of study ECGs for the detection of AF, which might have resulted in missing (paroxysmal) AF that was not present at the annual check-ups. This could potentially have led to underestimation of the association between PACs and AF if people with missed (paroxysmal) AF previously had PACs on a study ECG. By extension, this could also have led to underestimation of the association between PACs and BI, if antithrombotic medication was initiated in people with missed (paroxysmal) AF that had PACs on a study ECG. Another limitation is the use of mostly self-reported events. We could not distinguish ischemic from hemorrhagic stroke solely based and the self-reported cardiovascular morbidity. However, the self-reported cardiovascular morbidity registration was validated against hospital records in a sub-sample. In addition, we included only stroke events followed by antithrombotic medication use. Finally, the power of the separate TIA and ischemic stroke analyses is on the low side due to the limited number of events. This is reflected in the confidence intervals and increases the risk of a type II error. Therefore, the separate TIA and ischemic stroke analyses should be interpreted with caution.

## 5 Conclusions

In people with T2D without a history of AF or BI events, PACs (prevalent or incident) are associated with a two-fold increased risk of incident AF. Detection of a PAC should lead to the awareness that follow-up screening for AF might be prudent.

## Ethics declaration

This research was conducted with approval by the local medical ethical review board, and in accordance with the Helsinki Declaration on ethical principles for medical research involving human subjects.

## Funding

This work was supported by the 10.13039/501100001826Netherlands Organization for Health Research and Development (ZonMw) [80-83910-98-13046], the Dutch Heart Foundation grant CVON2017-15 RESCUED, the EFSD's European Pilot Research Grants for Innovative Measurement of Diabetes Outcomes, a grant from the Stichting Stoffels-Hoornstra charity foundation, and Amsterdam University Medical Centers.

The study funders were not involved in the design of the study; the collection, analysis, and interpretation of data or writing the report.

## CRediT authorship contribution statement

Jelle C.L. Himmelreich, Peter P. Harms, Marieke T. Blom, Joline W.J. Beulens, Giel Nijpels, Wim A.M. Lucassen, Petra Elders contributed to conception, design, data acquisition and analyses, interpretation of the results, review, and editing. Jelle C.L. Himmelreich and Peter P. Harms drafted the first manuscript and all authors reviewed the subsequent versions. All authors agreed to be accountable for aspects pertaining integrity or accuracy, and approved the final version. Jelle C.L. Himmelreich and Peter P. Harms are the guarantors of this work, and as such, had full access to all the data in the study and take responsibility for the integrity and the accuracy of the data analysis.

## Declaration of competing interest

The authors declare that they have no known competing financial interests or personal relationships that could have appeared to influence the work reported in this paper.

## Data Availability

The data that support the findings of this study are available from the Diabetes Care System (DCS) cohort steering committee, but restrictions apply to the availability of these data, which were used under license for the current study, and so are not publicly available. Data are, however, available from the authors upon reasonable request and with permission of the DCS cohort steering committee.

## Appendix A Supplementary data

Supplementary material Image 1
