# Supplementary material for: Longitudinal association of premature atrial contractions with atrial fibrillation and brain ischemia in people with type 2 diabetes: The Hoorn Diabetes Care System cohort
Source: Am Heart J Plus. 2023 Sep 6;34:100321. doi: 10.1016/j.ahjo.2023.100321 (PMC10945950; doi:10.1016/j.ahjo.2023.100321)
Supplement: Supplementary file 1 — Supplementary material [file mmc1.docx]

**Supplementary Material**

**Contents**

Table S1. The ECG abnormality categories used in this study based on aggregated Minnesota Classification codes.

Figure S2. Missing values of covariates at baseline and at subsequent follow-up measurements.

Table S3. Incidence rates with 95% confidence intervals for atrial fibrillation and brain ischemia events by ECG abnormality.

Table S1. The ECG abnormality categories used in this study based on aggregated Minnesota Classification codes.

| **ECG abnormality category** | **Minnesota Classification** | |
| --- | --- | --- |
|  | **code** | **definition** |
| Premature Atrial Contractions | 8-1-1 | *Defined in Minnesota Classification as*:  Presence of frequent atrial or junctional premature beats (10% or more of recorded complexes).  *Operationalised in Hoorn DCS cohort dataset as*:*  Presence of ≥1 premature atrial or junctional premature beats (in any or the recorded complexes) |
|  | 8-4-2 | Supraventricular tachycardia intermittent. Three consecutive atrial or junctional premature beats occurring at a rate ≥ 100. |
|  |  |  |
| Atrial Fibrillation | 8-3-1 | Atrial fibrillation. |
|  | 8-3-2 | Atrial flutter. |
|  | 8-3-3 | Intermittent atrial fibrillation (code if 3 or more clear-cut, consecutive sinus beats are present in any lead). |
|  | 8-3-4 | Intermittent atrial flutter (code of 3 or more clear-cut, consecutive sinus beats are present in any lead). |

* The ‘10% or more of recorded complexes’ criterion was not applied because it is generally not sensible for a 10 second ECG recording.

Note: During coding, no distinction was made between a few codes for two reasons.

1. differentiation between them was deemed clinically irrelevant:

- Atrial fibrillation or flutter (codes 8-3-1 and 8-3-2)).

1. because distinction between persistent and intermittent varieties of an ECG abnormality is generally impossible with a 10 second ECG recording:

- Atrial fibrillation (codes 8-3-1 and 8-3-3).
- Atrial flutter (codes 8-3-2 and 8-3-4).

Figure S2. Missing values of covariables at baseline and at subsequent follow-up measurements.

Data are presented as proportion (%). Baseline was defined as the first annual examination with an ECG recording after entry into the DCS cohort.

BMI, body mass index; SBP, systolic blood pressure; DBP, diastolic blood pressure; HbA1c, haemoglobin A1c; LDL, low density lipoprotein; HDL, high density lipoprotein; TC, total cholesterol; eGFR, estimated glomerular filtration rate; UACR, urinary albumin creatinine ratio; CVD: cardiovascular disease.

Table S3. Incidence rates with 95% confidence intervals for atrial fibrillation and brain ischemia events by ECG abnormality.

| **ECG abnormality category** | **AF** | **BI events** | **Time**  **(person years)** | **Incidence rate**  **(per 1000 person years)** |
| --- | --- | --- | --- | --- |
| **PAC** |  |  |  |  |
| no | 528 |  | 86788.4 | 6.1 (5.6-6.6) |
| yes | 111 |  | 5447.6 | 20.4 (16.8-24.5) |
| **PAC** |  |  |  |  |
| no |  | 470 | 88823.9 | 5.3 (4.8-5.8) |
| yes |  | 47 | 5865.4 | 8.0 (5.9-10.7) |
| **AF** |  |  |  |  |
| no |  | 496 | 92014.6 | 5.4 (4.9-5.9) |
| yes |  | 21 | 2674.7 | 7.9 (4.9-12.0) |

PAC: Premature atrial contractions, AF: Atrial Fibrillation, BI: Brain Ischemia.
